# Ultra-thin passivation layers in Cu(In,Ga)Se_2_ thin-film solar cells: full-area passivated front contacts and their impact on bulk doping

**DOI:** 10.1038/s41598-020-64448-9

**Published:** 2020-05-05

**Authors:** Florian Werner, Boris Veith-Wolf, Michele Melchiorre, Finn Babbe, Jan Schmidt, Susanne Siebentritt

**Affiliations:** 1 University of Luxembourg, Laboratory for Photovoltaics, Department of Physics and Materials Science, 41 rue du Brill, L-4422 Belvaux, Luxembourg; 2 Institute for Solar Energy Research Hamelin (ISFH), Am Ohrberg 1, D-31860 Emmerthal, Germany; 3 Department of Solar Energy, Institute of Solid-State Physics, Leibniz University Hannover, Appelstr. 2, D-30167 Hannover, Germany; 4 Present Address: Lawrence Berkeley Laboratory, Berkeley, USA

**Keywords:** Solar cells, Electronic properties and materials, Surfaces, interfaces and thin films

## Abstract

In the search for highly transparent and non-toxic alternative front layers replacing state-of-the-art CdS in Cu(In,Ga)Se_2_ thin-film solar cells, alternatives rarely exceed reference devices in terms of efficiency. Full-area ultra-thin aluminium oxide tunnelling layers do not require any contact patterning and thus overcome the main drawback of insulating passivation layers. Even a few monolayers of aluminium oxide can be deposited in a controlled manner by atomic layer deposition, they show excellent interface passivation properties, low absorption, and suitable current transport characteristics on test devices. Depositing a ZnO-based transparent front contact, however, results in extremely poor solar cell performance. The issue is not necessarily a low quality of the alternative front layer, but rather the intricate relation between front layer processing and electronic bulk properties in the absorber layer. We identify three challenges critical for the development of novel front passivation approaches: *(i)* both Cd and Zn impurities beneficially reduce the high native net dopant concentration in the space charge region, *(ii)* sputter deposition of ZnO damages the passivation layer resulting in increased interface recombination, *(iii)* thermal treatments of devices with ZnO layer result in substantial Zn diffusion, which can penetrate the full absorber thickness already at moderate temperatures.

## Introduction

**Figure 1:**
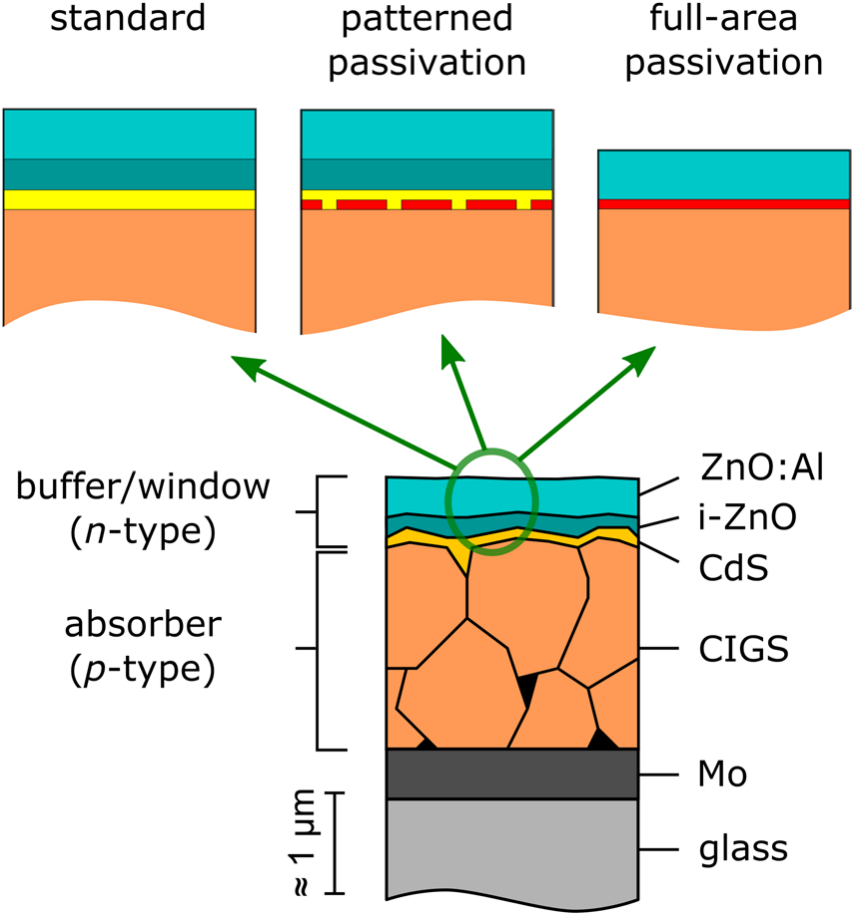
Device concepts. Sketch of a state-of-the-art CIGS thin-film solar cell (bottom) with soda-lime glass (SLG) substrate, Mo back contact, *p*-type polycrystalline CIGS absorber layer, and a standard *n*-type front buffer/window stack with CdS buffer layer, nominally intrinsic i-ZnO, and low-resistivity Al-doped ZnO. Three approaches for front-side engineering are sketched at the top: alternative (Cd-free) buffer layer (left), patterned dielectric passivation layer with a buffer layer passivating the contact openings (centre), and a full-area tunnelling passivation layer (right). High-bandgap dielectric passivation layer shown in red, low-bandgap conductive buffer layer shown in yellow.

Thin-film photovoltaic (PV) devices based on the ternary chalcopyrite Cu(In,Ga)Se_2_ (CIGS)^1–3^ are among the most efficient thin-film solar cells^4^, having demonstrated efficiencies of 20.8%^5^ on flexible and 23.35%^6,7^ on rigid substrates (22.3%^8^ for pure selenides containing no sulphur). A typical state-of-the-art CIGS thin-film solar cell is sketched in Fig. 1 and is made from a soda-lime glass (SLG) growth substrate, Mo back contact, *p*-type polycrystalline CIGS absorber layer deposited by vacuum co-deposition, and an *n*-type front buffer/window stack. This buffer/window stack forms the *n*-doped side of the *p/n*-heterojunction, passivates electronic defects at the front interface, and serves as transparent conductive front contact. The most widely used buffer/window stack consists of a CdS buffer layer deposited by chemical bath deposition, and a ZnO-based double window layer with nominally intrinsic i-ZnO and highly conductive Al-doped ZnO (AZO).

Despite the great success of the standard CdS + ZnO front stack so far, CdS has several drawbacks and represents a major limitation for future progress in CIGS thin-film PV:

- Cd is a toxic heavy metal, and besides the corresponding environmental and health considerations, its use could be restricted or banned by stricter policies.
- CdS has a fairly low optical bandgap around 2.4 eV resulting in significant parasitic absorption in the short-wavelength range^9^, thus reducing the short-circuit current density of the solar cell.

Alternative Cd-free buffer layer materials, which are still conductive but have a higher optical bandgap than CdS, and recently also insulating high-bandgap dielectric passivation layers are two potential approaches to overcome the limitations imposed by CdS.

Many alternative Cd-free buffer layers with higher optical bandgap than CdS have been studied and developed for CIGS thin-film PV, for example Zn(O,S,OH), Zn(Mg,O), In_2_S_3_, and many more^10–12^, and references therein. In most cases, however, alternative buffer layers successfully reduce parasitic absorption but fail to compete with the maximum efficiency and reliability of a conventional CdS buffer layer. Nevertheless, recent efficiency improvements leading to the current world-record Cu(In,Ga)(Se,S)_2_ solar cell with 23.35% efficiency were achieved with a Cd-free buffer layer^7^. Interestingly, the Zn-based buffer layer in these record devices results in a virtually identical short-circuit current density compared to the CdS reference, and instead increases the open-circuit voltage. This is partly ascribed to reduced non-radiative recombination, which the authors propose might be the result of switching from a CdS buffer layer to a Zn-based alternative preventing the formation of Cd-related defects inside the space charge region^7^. We recently demonstrated that a CdS buffer layer indeed alters the CIGS net dopant concentration, but found no significant changes in non-radiative recombination losses in our devices^13^. It becomes apparent that the effect of an alternative buffer layer on solar cell performance is much more complex and less understood than a simple consideration of absorption losses, interface passivation, and band alignment.

Insulating high-bandgap passivation layers – usually based on metal oxides or nitrides – are well-established in the field of crystalline silicon (c-Si) PV to efficiently suppress interface recombination^14,15^. Such metal oxide dielectric passivation layers have also been introduced to CIGS thin-film solar cells in recent years^16–20^ and are another promising alternative to replace the CdS buffer layer or reduce its thickness. Oxide-based passivation layers show excellent insulating properties, which complicates a straight-forward current extraction. The conventional approach is sketched in the upper centre of Fig. 1 and consists of patterning the passivation layer to open conductive pathways. This approach has several severe disadvantages in the case of CIGS:

- The low minority carrier diffusion length in polycrystalline CIGS of the order of a few micrometres or below requires structuring on micro- or nanoscale dimensions^21–24^.
- Patterning techniques applicable at these length scales, e.g., electron beam lithography, are not industrially viable for large-area processing.
- Etching the passivation layer might damage the CIGS surface inside the patterned contact openings^25^.
- Because the CIGS surface inside the contact openings is not protected and might be damaged during sputter deposition of the ZnO window layer, an additional buffer layer would still be needed to protect the contact openings.

Alternatively, passivation layers could be made thin enough to enable sufficiently high tunnelling currents without any contact patterning. Such a full-area tunnelling concept is shown in the upper right sketch of Fig. 1. In c-Si solar cells, ultra-thin AlO_*x*_ layers with a nominal thickness of only two monolayers have been used successfully as passivating tunnelling contacts between phosphorous-doped *n*^+^emitter and metal front contact^26^. Earlier results by Yousfi *et al*.^27^ on AlO_*x*_ deposited on CIGS suggest that a similar approach might also be feasible for CIGS. This is further supported by a slightly different approach by Keller *et al*.^28^ combining CdS with AlO_*x*_: because sub-nanometre-thin AlO_*x*_ covers pinholes in the CdS almost without impeding current extraction, good interface quality can be maintained with a reduced CdS thickness. In a fully passivated tunnelling approach, however, CdS would no longer be necessary at all.

Atomic layer deposition (ALD) is often the method of choice to deposit thin passivation layers on rough surfaces in thin-film devices. The principle of ALD is based on two alternating self-limiting half-reactions of different precursor molecules^29^, which ideally ensure that a highly conformal and compact layer forms monolayer by monolayer even on complex surface geometries. This property makes ALD highly relevant for the controlled deposition of thin films on rough interfaces, which is particularly important for the passivation of the rough front surface of polycrystalline CIGS absorber thin films. Notably, ALD has already been applied extensively in the last two decades in search for alternative Cd-free buffer layers on CIGS^10^.

In the present manuscript we propose that slow progress in the search for alternative Cd-free front passivation schemes is not necessarily due to unsuitable buffer layers, but in many cases might be related to so far unconsidered side-effects altering the CIGS bulk properties. We employ aluminium oxide deposited by plasma-assisted ALD as model system to study front interface passivation of polycrystalline CIGS solar cells. As introduced above, such ultra-thin tunnelling layers could elegantly replace CdS in a CIGS solar cell. In addition, they enable a precise fundamental electronic characterization of the CIGS absorber layer without any effects or artefacts potentially caused by the presence of typical buffer or window layers.

In this manuscript we study the growth process, passivation, and insulation properties of sub-nanometre-thin AlO_*x *_ tunnelling layers on rough polycrystalline CIGS surfaces. Note that throughout this manuscript we will use the notation “AlO_*x*_” to refer to (potentially off-stoichiometric) ultra-thin aluminium oxide layers of a few monolayers, to distinguish them from thicker (>5 nm) “bulk-like” Al_2_O_3_ layers. We employ such ultra-thin tunnelling layers in full-area front passivated solar cells, where we find that the excellent electronic quality of test devices cannot be transferred to real solar cells with a ZnO front contact layer. Because we suspect that ZnO deposition might play a major role in these performance issues, we study changes in the electronic CIGS bulk properties due to different front layers. We confirm a dramatic effect of Zn on bulk dopant concentration, which can be beneficial or detrimental depending on the magnitude of Zn migration. The penetration depth of Zn in CIGS in these experiments is surprisingly large, and at low temperatures certainly far exceeds any estimates based on simple diffusion models. We explain these discrepancies based on published diffusion experiments in literature, which revealed anomalous diffusion profiles due to interstitial migration within the vacancy-rich cupper sub-lattice in CIGS. We then discuss the fundamental limitations these interactions between absorber and buffer/window stack pose on potential future high-performance front stacks.

## Sub-nanometre AlO_*x*_ tunnel layers on CIGS surfaces

The use of Al_2_O_3_ passivation layers in CIGS thin-film PV is heavily inspired^19^ by its great success in the field of c-Si PV, and know-how from c-Si PV often guides the development of passivation concepts in thin-film PV. Nevertheless, caution is advised when drawing analogies between the two different technologies, in particular where interface chemistry and nucleation mechanisms are concerned.

ALD of Al_2_O_3_ on clean silicon surfaces has been found to require a few nucleation cycles to initiate the ALD process due to the unreactive hydrogen-terminated surface of c-Si^30^. These initial non-idealities result in a perturbed layer growth during the first 5–10 nm, which results in an incomplete passivation of interface trap states for Al_2_O_3_ passivation layers of a few nanometres or thinner^31,32^, but might in part be responsible for the formation of the negative fixed charge density at the c-Si/Al_2_O_3_ interface^32–34^. After this initial growth inhibition phase, a linear growth regime with a typical growth rate of 1.0–1.2 Å/cycle has been determined by spectral ellipsometry for Al_2_O_3_ layers deposited on c-Si^35^.

Results for c-Si cannot be compared directly to the present case of CIGS, however, because surface chemistry and morphology are very different between silicon and CIGS. Furthermore, the growth of nanometre-scale thin films on top of the rough polycrystalline CIGS surface is extremely challenging to observe directly with microscopy or ellipsometry, both due to the very thin AlO_*x*_ layers and the large surface roughness. Instead, we employ indirect measures – capacitance of metal-insulator-semiconductor (MIS) devices, attenuation of x-ray photoelectron spectroscopy (XPS) intensity, and current transport across a tunnelling barrier – to monitor the growth process of AlO_*x*_ on CIGS. These indirect estimates provide some hints confirming a controlled AlO_*x*_-ALD process also on rough CIGS surfaces, as discussed in the following.

### Insulator capacitance

**Figure 2:**
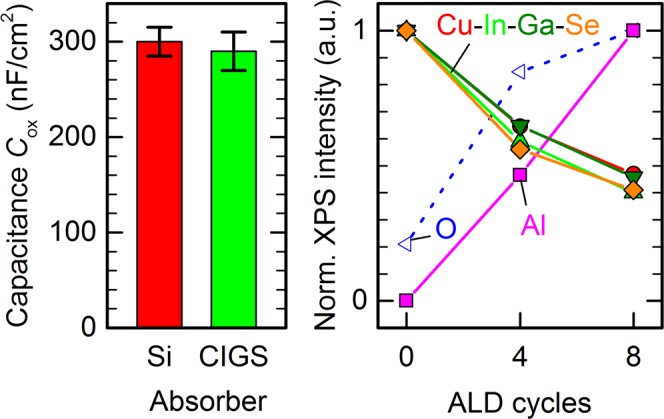
ALD on polycrystalline CIGS. Left: insulator capacitance *C*_ox_ of MIS devices with 180 ALD cycles of Al_2_O_3_ on c-Si and CIGS, respectively. Right: normalized XPS intensity of the relevant elements of a CIGS absorber covered with 0, 4, and 8 ALD cycles of AlO_*x*_: Cu 3p, In 3d, Ga d3, Se 3d peaks of CIGS normalized to the intensity for 0 cycles (bare etched absorber), and Al 2p and O 1s normalized to their intensities for 8 cycles.

For Au/Al_2_O_3_/CIGS metal-insulator-semiconductor (MIS) devices with 180 ALD cycles of Al_2_O_3_ at a substrate temperature of 130 °C, we measure insulator capacitances in the range of 290 ± 20 nF/cm^2^ as shown in Fig. 2. The reference process on c-Si wafers, 180 ALD cycles at a substrate temperature of 200 °C, results in Al/Al_2_O_3_/c-Si MIS devices with insulator capacitances of 300 ± 15 nF/cm^2^, and thus virtually identical to Al_2_O_3_ on CIGS. The physical thickness of the Al_2_O_3_ layer on c-Si is 21–22 nm, determined by spectral ellipsometry^35^, and we thus expect a similar thickness on CIGS. Note, however, that the relative dielectric permittivity ${\varepsilon }_{{\rm{r}}}$ of Al_2_O_3_ on c-Si is sensitive to the formation of a SiO_2_-like interlayer^35^, and the relation $d={\varepsilon }_{0}{\varepsilon }_{{\rm{r}}}/{C}_{{\rm{ox}}}$ between thickness *d* and insulator capacitance *C*_ox_ might be different for Al_2_O_3_ deposited on c-Si or CIGS.

### Attenuation of XPS intensity

We expect that the XPS intensities of the absorber elements – Cu, In, Ga, and Se – attenuate exponentially with increasing thicknesses of the growing AlO_*x*_ layer, while the Al and O intensity increases as more AlO_*x*_ material is present on the surface. This effect is shown in Fig. 2 for CIGS absorbers covered with 0, 4, or 8 ALD cycles of AlO_*x*_, respectively: the XPS intensity of absorber elements drops to 1/*e* after around 9–10 ALD cycles, indicating that the AlO_*x*_ layers grow by approximately one tenths of the inelastic mean free path (IMFP) per ALD cycle. For a more rigorous analysis it is important to note that the binding energy of the In 3d core level (444 eV) is much higher than that of Cu 3p (75 eV), Ga 3d (19.5 eV), or Se 3d (54 eV)^36^. The corresponding IMFP for photoelectrons emitted from In 3d levels is then expected to be lower, which explains why the In signal attenuates faster than that of Cu or Ga. In contrast to the other three absorber elements, we find less Se than expected from the considerations above. We attribute this Se deficiency to oxidation of the interface, which we described in a recent study^37^.

Using recent calculations for the energy dependence of the IMFP in crystalline Al_2_O_3_^38^, the Cu, In, and Ga peaks all yield the same growth rate of ALD-AlO_*x*_ of 2.5 ± 0.5 Å/cycle. This value can however only be a rough estimate due to the limited amount of data available, relying on a calculated IMFP for crystalline material, and because we neglect surface roughness. We expect from insulator capacitance measurements shown above that the actual growth rate is likely very similar to that on c-Si. The XPS studies nevertheless indicate, that ALD growth of AlO_*x*_ on CIGS likely proceeds as nearly ideal monolayer-by-monolayer growth even for only a few ALD cycles.

### Current density – voltage characteristics

These considerations above at least qualitatively indicate a controlled deposition of ultra-thin AlO_*x*_ also on rough CIGS surfaces, which demonstrates that AlO_*x*_ could in principle be used as tunnelling barrier for full-area passivated front contacts. Current density–voltage (*JV*) characteristics of MIS structures illustrate the current transport across a thin layer of AlO_*x*_, and thus provide further insight into the large-area spatial uniformity and compactness of the corresponding AlO_*x*_ film.

**Figure 3:**
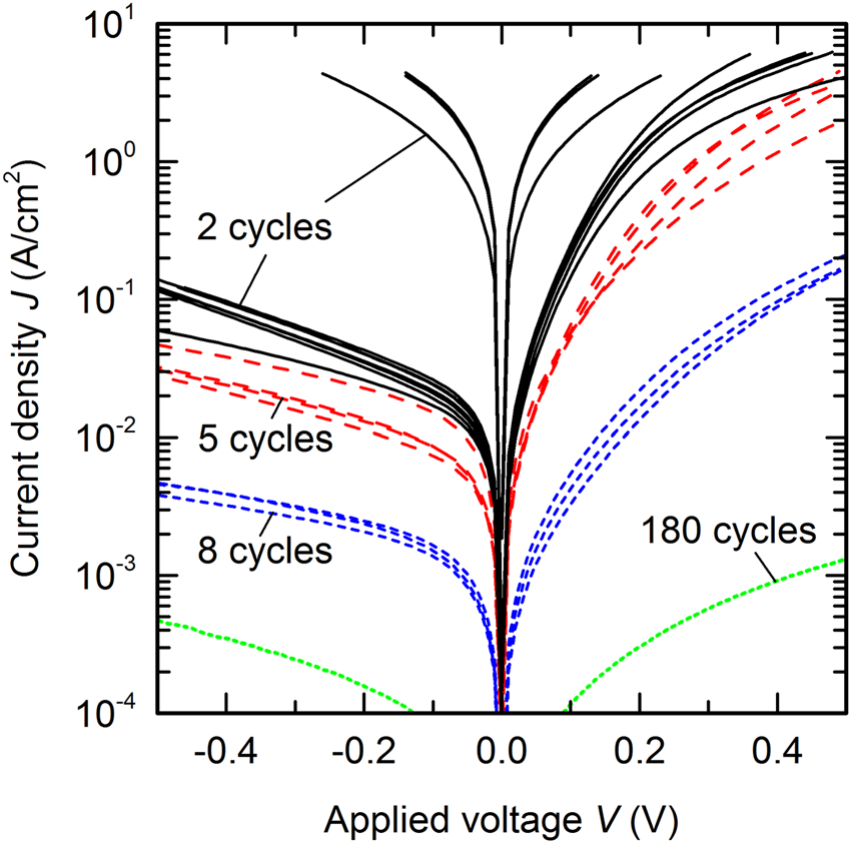
Current blocking of ultra-thin AlO_x_. Current density *J* as a function of voltage *V* at room temperature for as-deposited Au/AlO_*x*_/CIGS MIS devices with 2 (black solid lines), 5 (red long-dashed lines), 8 (blue short-dashed lines), and 180 (green dotted lines) ALD cycles.

Representative room-temperature *JV* characteristics are shown in Fig. 3 for several Au/AlO_*x*_/CIGS MIS devices fabricated from SLG/Mo/CIGS absorber with standard back contact, ultra-thin AlO_*x*_ layers with 2–8 ALD cycles, and circular Au front contacts with diameters of 1 mm. They exhibit a pronounced non-linear and asymmetric voltage dependence, which implies that current across the MIS devices must at least partly be ascribed to non-ohmic tunnelling or thermionic emission processes. Variations in *JV* characteristics for the same nominal thickness are likely related to inhomogeneities and small variations in actual thickness, and are more pronounced for thinner layers. Interestingly, only a few devices with the thinnest AlO_*x*_ layers – deposited with only 2 ALD cycles – result in nearly ohmic *JV* characteristics, which leads to two conclusions:

1. In most devices, 2 ALD cycles of AlO_*x*_ are indeed compact and insulating enough to impede current flow across the contacted area with a diameter of 1 mm. On a macroscopic scale, however, these extremely thin layers do not fully cover the surface and thus some devices contain holes in the AlO_*x*_, resulting in high and almost ohmic current flow.
2. Because current flow through pinholes in the AlO_*x*_ is nearly ohmic, we conclude that the Au top contact in the present work does not form a Schottky-type junction with CIGS. This confirms that any non-ohmic transport processes in the MIS devices must indeed be caused by the AlO_*x*_ layer, no matter how thin it might be.

From these capacitance, XPS, and *JV* measurements we conclude that the growth of AlO*x* on polycrystalline CIGS follows a nearly ideal ALD mode: each ALD cycle deposits one monolayer of AlO_*x*_ with a growth rate very similar to that on c-Si (1.0–1.2 Å/cycle). There is no significant growth inhibition phase, and already after 2 ALD cycles most of the sample surface is covered with an insulating layer. Throughout this paper we will use the number of ALD cycles to specify the thickness of each AlO_*x*_ layer. The physical thickness of such a layer will likely be very close to a nominal thickness defined by the number of ALD cycles multiplied by a typical growth rate of 1.0–1.2 Å/cycle.

### Passivated contacts

**Figure 4:**
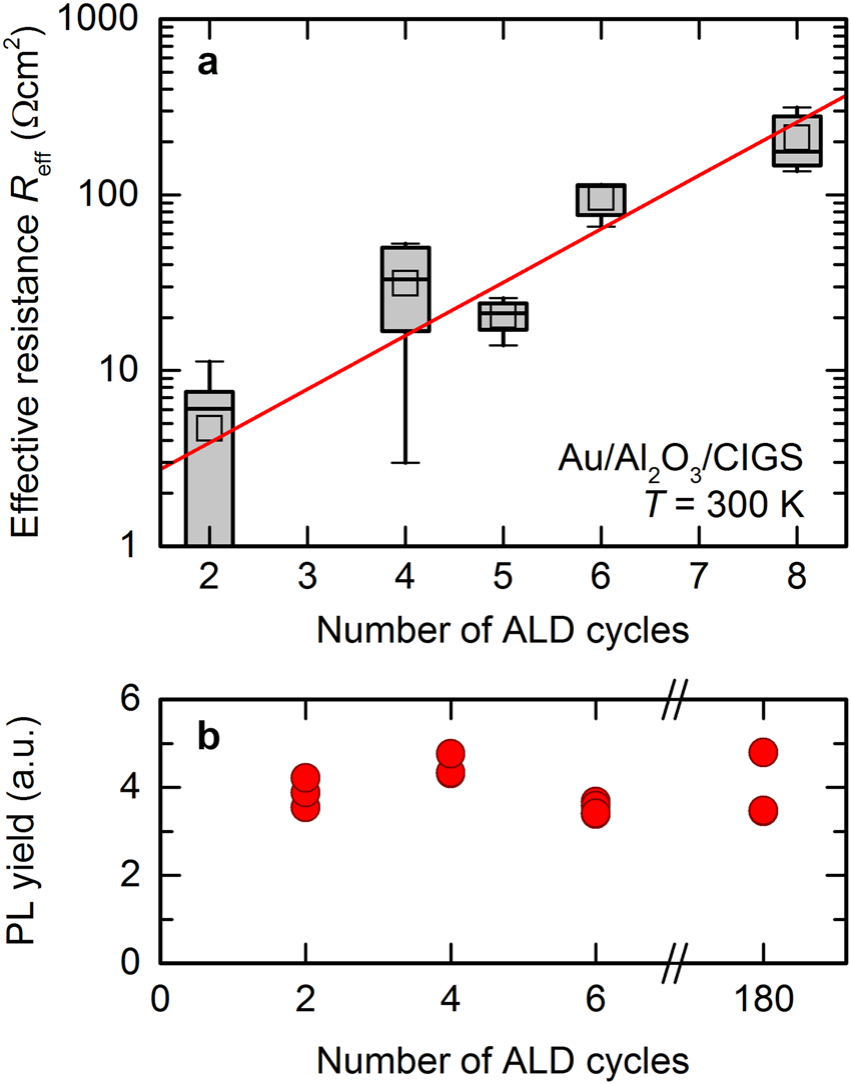
Passivated tunnel contacts. (**a**) Effective resistance *R*_eff_ = max(d*V*/d*J*) of Au/AlO_*x*_/CIGS MIS devices with ultra-thin AlO_*x*_ layers as a function of the number of ALD cycles. The layers studied here approximately cover a thickness range of 0.2–1.0 nm. The red solid line is an exponential fit. (**b**) Photoluminescence yield of CIGS/AlO_*x*_ devices annealed for 150 s at 350 °C for three sub-nanometre layers compared to 20 nm of Al_2_O_3_ (180 ALD cycles).

For a quantitative comparison of insulation properties, we define an effective resistance *R*_eff_ across the AlO_*x*_ layer near zero bias voltage by taking the maximum value of the slope d*V*/d*J* of a given experimental *JV* curve. We consider this maximum slope most representative of the resistance of the AlO_*x*_ layer itself, since it minimizes the effect of field-assisted transport processes. Note that the maximum in the slope d*V*/d*J* for our samples typically occurs between voltages of 0 V and up to a few 100 mV in reverse bias. We attribute these small deviations from zero bias to built-in potentials due to charges at the AlO_*x*_/CIGS interface. Figure 4(a) shows a boxplot of the distribution of effective resistance values as a function of the number of ALD cycles used in the deposition process. For each nominal thickness, we measure 4 different spots on two samples processed in different runs several months apart. We observe a large spread in resistance for the thinnest layers (2 and 4 ALD cycles), which indicates that these AlO_*x*_ layers are likely not homogenous on a millimetre or centimetre scale. In particular, several individual measurements for the thinnest layers with 2 ALD cycles yield drastically reduced effective series resistance values, suggesting the presence of pin-holes within the AlO_*x*_ or even larger uncovered patches. Neglecting these outliers, the effective resistance increases exponentially with the number of ALD cycles, as indicated by the exponential fit (red line) in Fig. 4(a). We expect the tunnelling probability to decrease exponentially with insulator thickness, and thus conclude that the actual physical thickness of the AlO_*x*_ layers is proportional to the nominal thickness given by the number of ALD cycles during deposition. Accordingly, ALD of AlO_*x*_ on CIGS appears to result in an approximately linear growth, i.e., constant growth rate, starting from the very first cycle.

While 2 ALD cycles appear to be too thin to fully cover the CIGS surface over large areas, the exponential increase of effective resistance means that any full-area (i.e., without artificially introduced contact openings) AlO_*x*_ passivation layer within a solar cell must only be a few monolayers thin to permit sufficient current flow across the insulator.

Our electrical measurements confirm that it is indeed possible to deposit compact layers of AlO_*x*_ with sub-nanometre thickness by ALD on rough polycrystalline CIGS surfaces, but do not reveal whether such thin layers in fact still passivate the CIGS surface. Figure 4(b) shows the photoluminescence (PL) yield of several CIGS absorbers passivated with sub-nanometre AlO_*x*_ layers (2, 4, and 6 ALD cycles) compared to a “thick” Al_2_O_3_ layer of nominally 20 nm (180 ALD cycles). All devices have been annealed for 150 s at 350 °C in nitrogen ambient to activate the passivation^37^. The PL yield depends *exponentially* on quasi-Fermi level splitting, and small differences in PL yield are thus negligible. Accordingly, even the thinnest layers with only 2 ALD cycles afford a similar level of surface passivation as thicker layers. The PL yield has been remeasured several times over a period of one year storage in vacuum boxes, and the passivation quality for all samples remained stable.

## Solar cells with full-area front passivation

A full-area *n*-type transparent conductive front contact on top of the ultra-thin AlO_*x*_ tunnel passivation is required to fabricate full solar cells, replacing the circular opaque Au contacts in MIS devices. We use a sputtered aluminium-doped ZnO (AZO) window layer with Ni/Al front grid. Note that we employ a single-layer AZO front window instead of the standard double layer of nominally intrinsic i-ZnO and highly-doped AZO, because we expect that the resistance across the full-area passivation layer will make the resistive i-ZnO redundant. Front-passivated devices also do not contain any CdS, because the passivation layer is intended to fully replace the buffer layer.

We find that 2 ALD cycles of AlO_*x*_ as front passivation result in highly shunted devices, in agreement with the low effective resistance shown in Fig. 4, presumably because direct contact between AZO and CIGS at pinholes in the passivation layers results in poor junction quality. Front passivation layers with 4 ALD cycles or more exhibit no significant shunting, and instead, as expected, show an increasing severe current blocking with increasing thickness of the AlO_*x*_ layer. In the following discussion we exemplarily present a device with 5 ALD cycles of AlO_*x*_, corresponding to a nominal thickness of 0.5–0.6 nm. We find that this choice represents the best compromise to avoid shunting issues (thinner layers) and severe current blocking (thicker layers). The general trend, however, is similar for other sub-nanometre-thicknesses of AlO_x_ as well.

**Figure 5:**
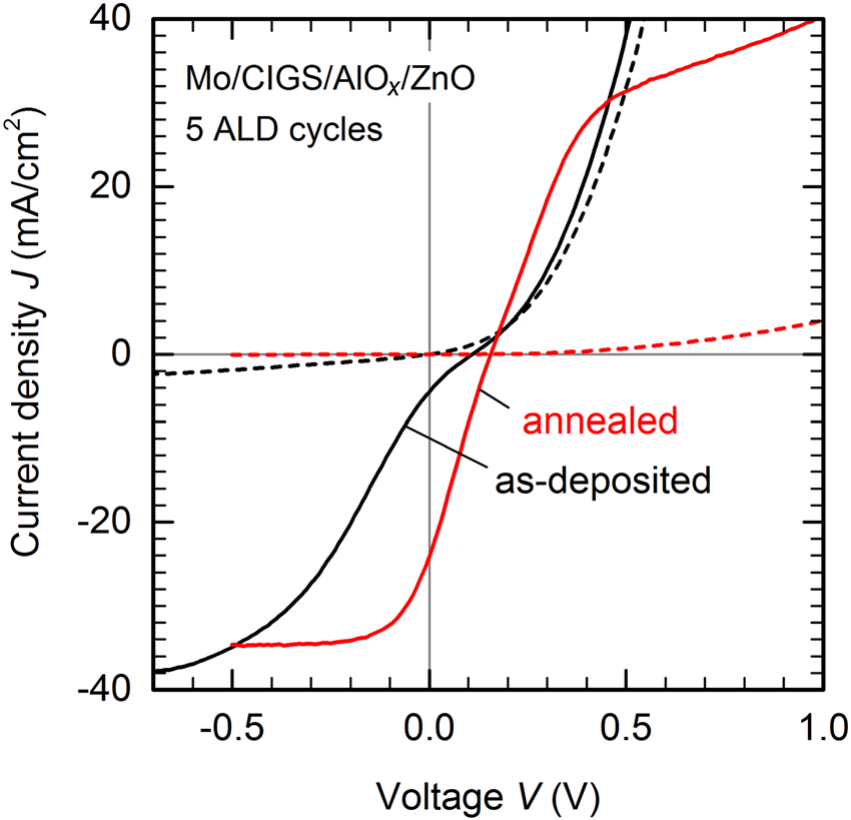
Front-passivated solar cells. Dark (dashed lines) and illuminated (solid lines) current density – voltage (*JV*) characteristics of a Mo/CIGS/AlO_*x*_/ZnO solar cell with 5 ALD cycles of AlO_*x*_ as front tunnel passivation layer: as-deposited (black) and after annealing for 60 s at 350 °C in nitrogen ambient (red).

Black lines in Fig. 5 show dark and illuminated *JV* characteristics for a full-area front passivation layer of 5 ALD cycles of AlO_*x*_. Despite the promising optoelectronic properties of the MIS test devices presented above, the resulting solar cell performance is very poor concerning all relevant parameters: open-circuit voltage *V*_oc_, short-circuit current density *J*_sc_, and fill factor *FF*. While we retain carrier selective transport, evidenced by the highly asymmetric dark *JV* curve, the built-in voltage is only a few 100 meV. And while the total photo-generated current density of 35 mA/cm^2^ at −0.7 V bias is in principle competitive, a significant photocurrent can only be extracted in reverse bias. This indicates that no driving force for current extraction in moderate forward bias exists in these front-passivated devices, and as a consequence *J*_sc_ is nearly zero. Even if no such extraction barrier was present, low *V*_oc_ and *FF* would limit this device to efficiencies around 8%, estimated by shifting the dark *JV* curve by 35 mA/cm^2^ corresponding to the estimated maximum *J*_sc_. Four devices each were fabricated for different thicknesses of AlO_*x*_, and all show the same behaviour. Performance remains low during storage in vacuum or air, and also does not improve under illumination at room temperature, indicating that the low performance is related to a fundamental issue with front-passivated CIGS solar cells.

Recombination via interface defects might partly be responsible for the poor solar cell performance.We previously demonstrated that thermal post-deposition annealing of AlO_*x*_-passivated absorber layers fully removes interface defects created during the ALD process^37^. Nevertheless, admittance spectroscopy (see Supplementary Figs. S1 and S2) reveals the presence of electronic interface traps in CIGS/AlO_*x*_/AZO solar cells, even if the AlO_*x*_-passivated absorber was annealed before AZO deposition to recover any interfacial damage introduced during AlO_*x*_ deposition. Accordingly, we conclude that the AZO deposition process by rf-sputtering itself is harmful for ultra-thin passivation layers, and notably damages the CIGS/AlO_*x*_ interface. Indeed, if the thickness of the AlO_*x*_ passivation layer is increased from 5 to 8 ALD cycles, the built-in voltage increases significantly (see Supplementary Fig. S3), presumably because a thicker passivation layer provides better protection of the CIGS surface during ZnO sputtering. Note that an actual open-circuit voltage cannot be determined in that case, because the photocurrent is essentially zero due to the high resistivity of the thicker AlO_*x*_ layer. Independent of the initial passivation quality of the front passivation layer, the AZO deposition process would ultimately result in a highly damaged front interface if the passivation layer is not thick enough to protect the CIGS surface from the AZO sputtering process.

A softer deposition process for the AZO transparent front contact could potentially solve this stability issue, but is undesirable because a long-established and cheap standard sputtering process would likely need to be replaced by less established technologies. Alternatively, it stands to reason that thermal annealing after AZO deposition could succeed in removing any detrimental interface traps created during the sputtering process, since it is also very effective to recover interface damage created during ALD of the passivation layer. Surprisingly, such a treatment results in drastic changes in the fundamental electronic properties, as shown by the red lines in Fig. 5 after annealing a front-passivated solar cell for 60 s at 350 °C. The solar cell becomes essentially non-conducting in the dark, and the photocurrent under illumination changes sign with applied voltage. Note that the illuminated *JV* characteristics are almost symmetric around a built-in potential of 160 mV. Such behaviour would be expected for an intrinsic semiconductor sandwiched between metal-like contacts. We confirm such a model by capacitance measurements, which are discussed in detail in the following Section. With standard front contact processes, the open-circuit voltage potential of full-area AlO_*x*_-front-passivated devices is thus limited either by the ability to deposit all front layer virtually defect-free, or by the thermal budget resulting in destruction of the basic device architecture during a post-fabrication thermal annealing.

**Figure 6:**
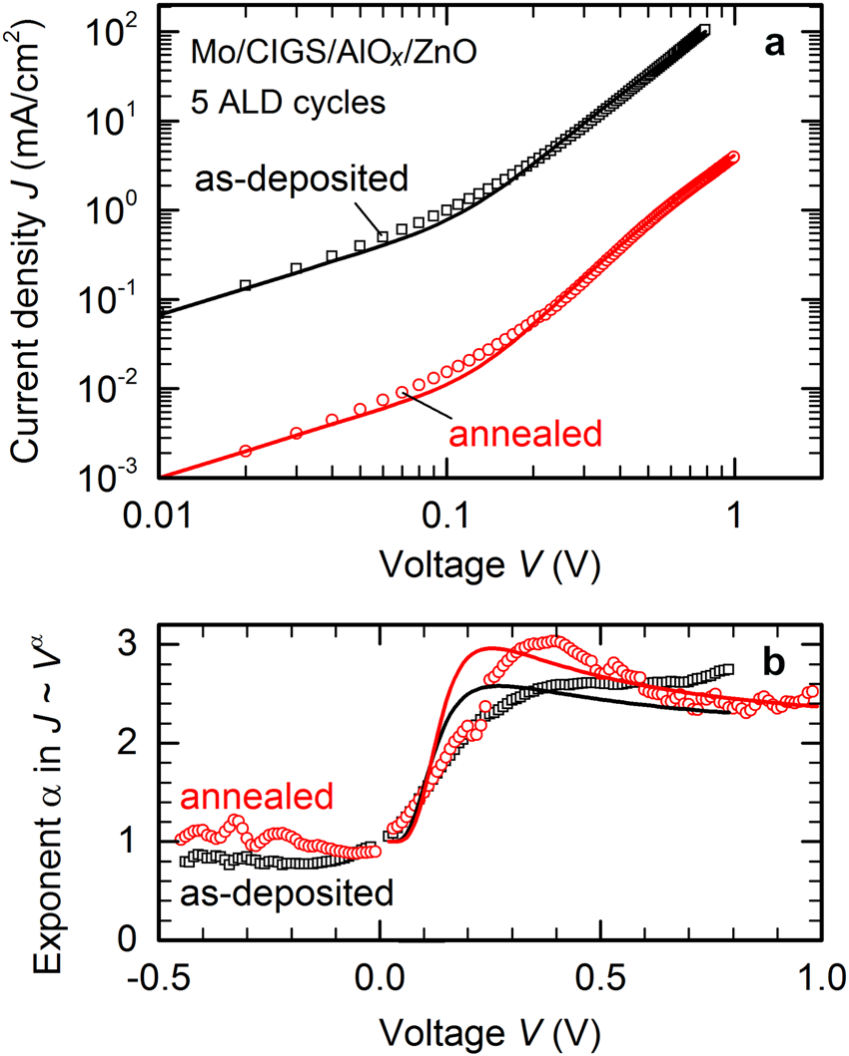
Electric transport model. (**a**) Dark *JV* characteristics of the devices shown in Fig. 5 replotted in double-logarithmic scale (open symbols) and fit to a tunnelling or field emission transport model, Eq. (1) (solid lines). (**b**) Local exponent α of a power-law voltage dependence $J\propto {V}^{\alpha }$ for experimental data and fit shown in (**a**).

Another fundamental limitation for the application of tunnelling contacts arises from the low *FF*, which is ultimately linked to a non-exponential current-voltage relation evident from the double-logarithmic plot of dark *JV* characteristics shown in Fig. 6. Field emission or tunnelling across a thin dielectric layer can be described by an approximately *quadratic* voltage-dependence of the current density or, more precisely, by the relation^[^^39^^, page 403]^

1 $$J(V)={J}_{0}{V}^{2}\exp (-b/V)+\frac{V}{{R}_{{\rm{p}}}}$$

with constants *J*_0_ and *b*, including a parasitic shunt resistance *R*_p_. Solid lines in Fig. 6 are fits of Eq. (1) to experimental dark *JV* characteristics of the front-side passivated solar cells shown in Fig. 5, and match the experimental voltage dependence reasonably well. Fit parameters are *J*_0_ = 220 mAcm^−2^V^−2^, *b* = 0.3 V, *R*_p_ = 150 Ωcm^2^ for the as-deposited device, and *J*_0_ = 6 mAcm^−2^V^−2^, *b* = 0.4 V, *R*_p_ = 10 kΩcm^2^ after annealing. A comparison of the local exponent α of a power-law voltage dependence $J\propto {V}^{\alpha }$ in Fig. 6(b) – or in other words, the slope of the double-log plot in Fig. 6(a) – shows that the tunneling models reproduces the qualitative shape of the experimental *JV* characteristics suitably well. Accordingly, the dark current is driven by tunnelling and shunt currents and shows little evidence of a space charge region. Upon annealing, the tunnelling barrier – contained in the parameter *b* – essentially remains the same, but the overall resistance increases dramatically.

## Influence of the ZnO front contact on electronic bulk properties

Thermal annealing of CIGS devices containing an AZO front layer appears to have a dramatic impact on electronic bulk properties, most prominently on the concentration of free carriers in the CIGS absorber bulk, which can be seen already from the *JV* characteristics shown in Figs. 5 and 6. This bulk effect is in addition to potential modifications of the interfacial region between CIGS and passivation layer or buffer/window stack. As we will discuss in the following, such drastic bulk changes are related to elemental inter-diffusion and resulting changes in net dopant concentration.

In a number of recent publications^13,40,41^ we discussed the correct interpretation of apparent doping profiles in thin-film solar cells, where we found that the deposition of a front layer has a dramatic influence on the doping profile of the underlying CIGS absorber. For Cu-poor CIGS we found that Hall measurement of bare absorbers^40^ always yield carrier concentrations higher than expected from capacitance-voltage (*CV*) measurement of full solar cells. In ref. ^41^ we demonstrated that doping profiles from *CV* are consistent with Cd diffusion from the CdS buffer layer into Cu vacancies inside the CIGS, and that these diffusion processes proceed even at room temperature. Accordingly, experimental doping profiles depend critically on the chemical composition – and thus number of Cu vacancies – of the absorber, the properties of the buffer layer, and on the age of the device due to continuing diffusion during storage. We proposed that Cd migrates into Cu vacancies in CIGS and reduces the net doping concentration, both by forming shallow substitutional donors (Cd_Cu_)^42–44^ and by removing shallow acceptors by filling Cu vacancies (V_Cu_), which are considered to be the dominant acceptor-type defect in CIGS^45–48^. In ref. ^13^ we found the same effect also for Cd-free Zn(O,S) buffer layers. In fact, both CdS/ZnO and Zn(O,S)/ZnO stacks on CIGS result in very similar bulk doping profiles, and the reduction of surface-near net doping due to in-diffusion appears to be stronger with a full stack than for CdS alone (compare Fig. 5 in ref. ^13^). Combined with the drastic changes upon annealing of Cd-free front-passivated solar cells shown in the previous Section, this suggest that also ZnO plays a crucial role in defining the electronic bulk properties of CIGS solar cells. The isoelectronic elements Cd and Zn in fact behave quite similar with respect to defect physics, and just like Cd_Cu_ the corresponding Zn_Cu_ is predicted to form a shallow donor in CIGS as well^43^.

Changes within the CIGS absorber of a standard thin-film solar cell due to impurity (e.g., Cd or Zn) migration are difficult to observe directly, because typical net dopant concentrations of 10^17^ cm^−3^ or below correspond to fewer than 10 free carriers per one million atoms in the crystal lattice. While large impurity concentrations can be detected reliably – e.g., by secondary ion mass spectrometry (SIMS), atom probe tomography (ATP), or energy-dispersive x-ray (EDX) – it is often not possible to exclude the presence of parts-per-million trace concentrations, which are below the experimental detection limit but still comparable to or larger than the net dopant concentration.

A systematic indirect observation of elemental diffusion by measuring its electronic effect on doping concentration, e.g., by *CV* measurements, is hindered by the fact that Cd- or Zn-containing front layers are usually always present to form the *p/n*-junction in a representative thin-film solar cell, making it difficult to obtain Cd- and Zn-free comparable reference devices. We find that Al_2_O_3_ passivation layers, if grown thick enough, form an excellent diffusion barrier for Zn impurities (see below), and thus allow to study the CIGS absorber layer independent of any Cd or Zn contamination. Furthermore, the good insulating properties of Al_2_O_3_ and the excellent control over electronic interface states allow precise measurements in a metal-insulator-semiconductor (MIS) configuration.

**Figure 7:**
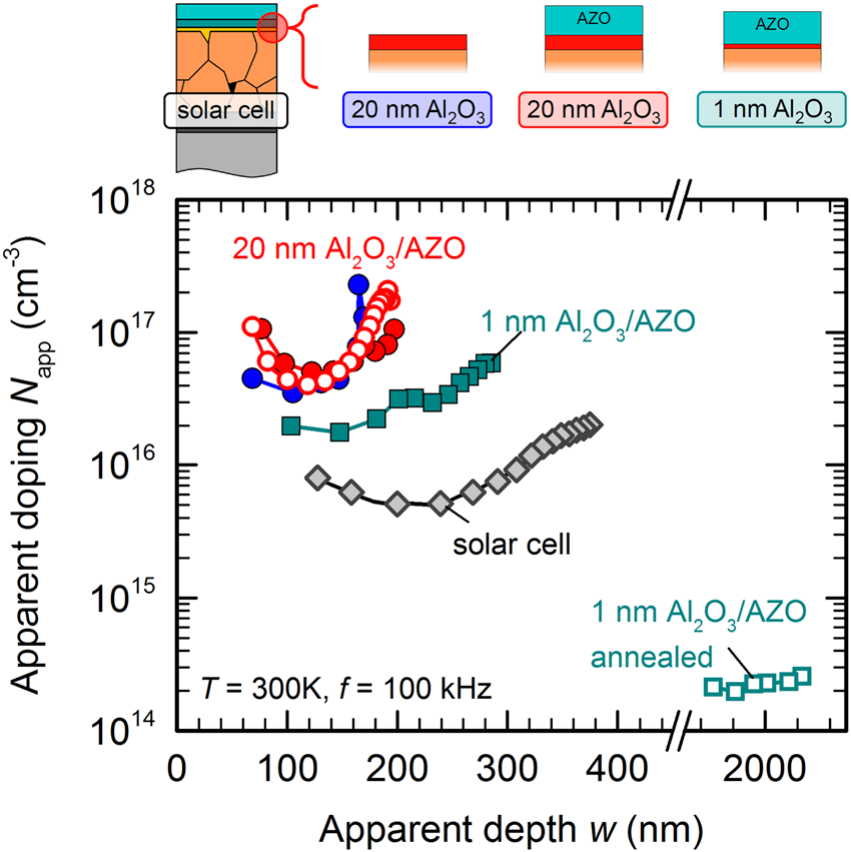
Doping effect by inter-diffusion. Sketch (top) and experimental apparent depth-dependent doping profiles *N*_app_(*w*) determined by CV measurements (bottom) of SLG/Mo/CIGS devices with four different front stacks: a solar cell with standard CdS/i-ZnO/AZO buffer/window stack (grey), 20 nm of Al_2_O_3_ (blue), 20 nm of Al_2_O_3_ covered with AZO (red), and ≈1 nm of Al_2_O_3_ covered with AZO (green). Open symbols report the dopant concentration after the full device had been annealed for 60 s at 350 °C in nitrogen.

Figure 7 shows experimental depth-dependent apparent doping profiles *N*_app_(*w*) at *T* = 300 K for different annealing conditions and different device structures sketched above the main figure: A standard solar cell with CdS/i-ZnO/AZO front stack (grey diamonds), and the same CIGS absorber with either 20 nm thick Al_2_O_3_ (blue circles), 20 nm thick Al_2_O_3_ covered with AZO (red circles), or approximately 1 nm AlO_*x*_ covered with AZO (green squares), respectively. All devices share CIGS absorber layers from the same deposition run and have Ni/Al front contacts.

Closed symbols in Fig. 7 show measurements on as-deposited devices without any thermal post-deposition treatments. The standard solar cell shows a typical doping profile for a CIGS solar cell^13,49–52^ with dopant concentrations around mid-10^15^ to 10^16^ cm^−3^. MIS devices with “thick” (20 nm) Al_2_O_3_ (blue and red circles) show higher average dopant concentrations slightly below 10^17^ cm^−3^ both with and without an additional AZO layer on top. Such high dopant concentrations, around one order of magnitude higher than found in solar cells, are in good agreement with earlier Hall measurements^40,41^ of the native bulk dopant concentration in pristine polycrystalline CIGS thin films. In accordance with ref. ^13^ we attribute the lower dopant concentration in solar cell devices to the electronic effect of Cd or Zn in-diffusion. Thick Al_2_O_3_ apparently prevents any Zn diffusion from the AZO front layer into the CIGS, and the CIGS/Al_2_O_3_ and CIGS/Al_2_O_3_/AZO devices both reveal the same native bulk doping unaffected by Zn diffusion. If the Al_2_O_3_ layer is too thin, however, in particular in the sub-nanometre regime relevant for full-area tunnel passivation layers, it can no longer fully prevent Zn migration. With approximately 1 nm AlO_*x*_ between CIGS and AZO (green squares in Fig. 7), the net bulk doping inside the CIGS absorber is noticeably reduced already for the as-deposited device.

Temperature is another critical parameter besides insulator thickness. An intermediate annealing step after Al_2_O_3_ deposition – but *before* sputter-deposition of AZO – improves the interface quality but leaves the net doping concentration within the CIGS essentially unchanged^37^. In contrast, annealing *after* AZO deposition can have a drastic impact on the bulk net dopant concentration, at least if Zn diffusion is not suppressed by a sufficiently thick Al_2_O_3_ diffusion barrier. Open symbols in Fig. 7 represent measurements after annealing the full device after AZO deposition for 1 min at 350 °C in nitrogen ambient in a rapid thermal processing furnace. With 20 nm of Al_2_O_3_ separating CIGS from the AZO front layer (red circles), annealing has no effect on the dopant concentration. We conclude that such thick Al_2_O_3_ layers remain excellent diffusion barriers for Zn even at elevated temperature. With only 1 nm of AlO_*x*_, where already the as-deposited device revealed minor electronic changes due to Zn diffusion, annealing after AZO deposition drastically enhances Zn migration into CIGS. The experimental capacitance value measured after annealing is comparable to the geometric capacitance *C*_geo_ of the CIGS absorber layer (${C}_{{\rm{geo}}}={\varepsilon }_{0}{\varepsilon }_{{\rm{r}}}/d$, with *d* ≈ 2 μm the physical thickness of the semiconducting absorber layer). An experimental capacitance close to the geometric capacitance is a clear indication that the free carrier concentration in the absorber layer is very low, i.e., the CIGS absorber is almost fully depleted. The corresponding doping profile extracted from the voltage-dependent *CV* measurement is reported in Fig. 7 and shows very low apparent dopant concentrations of about 10^14^ cm^−3^. Such low charge densities – and correspondingly small capacitances – are however highly susceptible to parasitic capacitances abundant in measurements of polycrystalline thin-film devices^53^, and the actual dopant concentration might be even lower and close to zero. An (almost) fully depleted absorber with extremely low free carrier concentrations is also in excellent agreement with the nearly symmetric current-voltage characteristics of annealed front-passivated devices shown in Fig. 5.

Pronounced changes in bulk dopant concentration after annealing in the presence of a ZnO front layer confirm the strong electronic effect of elemental interdiffusion on CIGS bulk properties. Because our MIS samples do not contain a CdS layer, we further confirm that Zn-containing compounds – at least qualitatively – have the same effect on CIGS doping as Cd-containing compounds. It is compelling to assume that the main electronic effect stems from Zn or Cd diffusing *into* CIGS. Instead, or in addition, mobile elements like Cu or Na might diffuse *out of* the CIGS layer. Annealing a CIGS thin film passivated with Al_2_O_3_ indeed leads to Na accumulation and Cu depletion from the CIGS/Al_2_O_3_ interface, as we reported previously^37^. This rearrangement of the absorber constituents, however, appears to be limited to a small surface-near region, and leaves the dopant concentration in the bulk of the absorber essentially unchanged. Furthermore, Cu migration out of the absorber layer would increase the concentration of acceptor-type Cu vacancies in CIGS and thus likely *increase* the net dopant concentration, opposite to the reduced net doping found in experiment. Na, on the other hand, typically increases *p*-type doping in CIGS^48,54^, and references therein, and loss of Na from the CIGS absorber could potentially explain a reduced net doping. In the present case, significant loss of Na from the absorber seems unlikely: the Na accumulation at the front interface of Al_2_O_3_-passivated CIGS is compensated by additional Na supply from the SLG substrate and does not lead to Na depletion in the bulk (see Supplementary Fig. S4). We also exclude oxygen as relevant species, because oxygen is readily available in Al_2_O_3_ where we observe no changes in bulk dopant concentration.

With Cu, O, or alkali elements unlikely to have a major effect on the bulk dopant changes observed in experiment, diffusion of Zn into the CIGS layer indeed remains the most plausible explanation.

## Expected Zn diffusion depth from literature data

For Zn atoms to meaningfully affect macroscopic electronic properties throughout the absorber bulk, they must be sufficiently mobile within CIGS. Nishiwaki *et al*.^55^ reported Zn depth profiles for Zn-doped polycrystalline CIGS absorbers, where evaporated metallic Zn was used as diffusion source in an *in-situ* physical vapor deposition (PVD) process at substrate temperatures of 100–400 °C. Their approach results in Zn concentrations far exceeding the dopant concentration by several orders of magnitude ([Zn] >> 10^19^ atoms/cm^3^) at 300 °C or above, with diffusion depths approaching 1 µm at 400 °C. While they find that Zn diffusion at 100 and 200 °C is negligible, the background noise in their depth profiles corresponds to Zn concentrations of approximately 10^19^ cm^−3^ (Fig. 5 in ref. ^55^), far too high to exclude a doping effect in CIGS. At higher temperatures, their measurements clearly support a substantial migration of Zn into the CIGS absorber.

**Figure 8:**
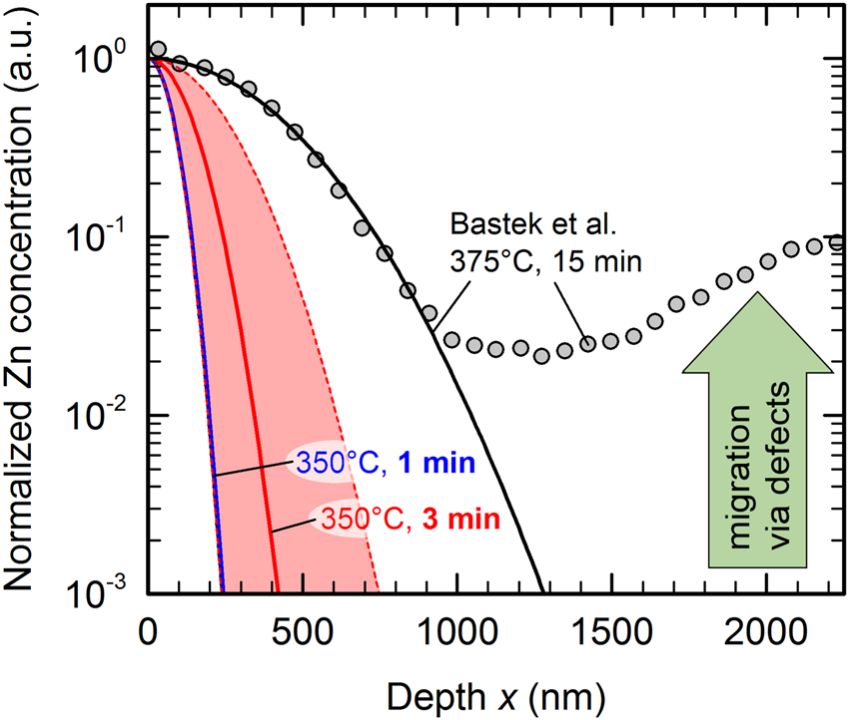
Zinc diffusion in CIGS. Literature data of experimental Zn diffusion profile digitized from Bastek *et al*. (Fig. 1 in ref. ^56^, grey symbols, diffusion treatment for 15 min at 375 °C). Based on diffusion coefficients from the same publication^56^, solid lines show the expected diffusion profiles for their experiment (black line) and for annealing at 350 °C for 1 min (blue) or 3 min (red), respectively. The shaded red area indicates the uncertainty in the diffusion coefficient and its extrapolation to 350 °C.

Radiotracer diffusion experiments by Bastek *et al*.^56^ provide quantitative parameters for the temperature-dependent diffusivity of Zn in polycrystalline CIGS. These values enable us to estimate whether substantial Zn diffusion over hundreds of nanometers or even some micrometers of CIGS is plausible in our *CV* experiments. For comparison, gray symbols in Fig. 8 quote an experimental Zn depth profile measured by Bastek *et al*. after a 15 min diffusion treatment at 375 °C (compare Fig. 1 in ref. ^56^), together with the theoretical Gaussian diffusion profile calculated from the experimental diffusion coefficient given in the same publication.

Assuming that every Zn atom in CIGS is electronically active, fully compensating 10^17^ cm^−3^ acceptors out of approximately 4 × 10^22^ cm^−3^ atoms in CIGS only requires a few 10^–4^ at% – or a few parts per million – of Zn inside the CIGS absorber. Bastek *et al*. have estimated the surface peak concentration of Zn in their experiment to be around 0.2 at%, in which case a normalized Zn concentration around 10^–3^ would just be enough to fully compensate the CIGS bulk doping. The calculated diffusion profile after annealing for 60 s at 350 °C (blue line in Fig. 8) suggests that significant acceptor compensation should only occur within the first few hundred nanometres. However, if additional heating and cooling times (represented exemplarily by an effective duration of 3 min at 350 °C, red line in Fig. 8) or uncertainties in the experimental diffusion coefficient (red shaded area, reported uncertainties^56^ in activation energy and prefactor) are taken into account, we find that Zn diffusion could indeed potentially affect a significant fraction of the full absorber thickness.

Much more importantly, however, Bastek *et al*. found that Zn diffusion follows a Gaussian profile only near the front surface, while Zn concentrations deep within the bulk and near the back contact are much higher than expected (see Fig. 8). Surprisingly, this is also the case in single-crystalline CuInSe_2_, and thus not related to grain boundary diffusion^56^. Migration of Zn via point defects, for example cupper vacancies, could explain such anomalous diffusion profiles^56^. Because typical Cu-poor CIGS solar cell absorbers contain very large quantities of cupper vacancies of the order of atomic percent^57^, we must assume that parts-per-million-quantities of Zn can easily migrate throughout the absorber layer even at temperatures well below the 350 °C studied here. Such defect-mediated Zn migration can also explain the typical doping profiles of CIGS solar cells with standard CdS buffer layer (grey diamonds in Fig. 7) or with alternative ultra-thin passivation layers (green squares in Fig. 7): although the substrate temperature likely does not exceed 100 °C during processing of the front layer stack, Cd or Zn impurities can migrate via Cu vacancies much further than expected from an idealized Gaussian diffusion profile based on reported diffusion coefficients. As a result, the net dopant concentration near the front of the CIGS absorber is significantly reduced over hundreds of nanometres. Note that these diffusion processes continue even at room temperature^41^, potentially modifying electronic device properties continuously throughout the lifetime of the solar cell.

Accordingly, Cd and Zn migration and its impact on electronic bulk properties must be taken into account whenever different front layers or heat treatments are applied to a solar cell, or whenever long-term stability is concerned.

## Relevance for the development of alternative front layers

Novel concepts – like dielectric surface passivation layers in thin-film photovoltaics – are often studied either in isolation on dedicated test samples, or on device level in a fully processed solar cell. Our results discussed in the previous Sections clearly show that modifying one constituent in the device architecture significantly affects other parts of the device. On the one hand, test devices that do not see the full fabrication process are not representative for full devices, because later processing steps can significantly alter any layer in the complex device architecture. On the other hand, the poor performance of a fully processed solar cell – in our case with full-area AlO_*x*_ tunnel passivation layer – does not necessarily indicate a low quality of the AlO_*x*_ tunnel layer, which had demonstrated excellent potential without the AZO front contact. We find that the issue is not necessarily a low quality of the alternative front layer, in our case AlO_*x*_, but rather a consequence of the intricate relation between front layer processing and electronic bulk properties inside the CIGS absorber itself.

Changes in interface state density and net dopant concentration inside the SCR explain the low efficiencies of AlO_*x*_ full-area front-passivated solar cells, and could also explain the poor performance of many alternative front-side passivation or buffer layers. The ALD process during Al_2_O_3_ deposition creates interface defects, which can be healed by a suitable post-deposition anneal^37^. In the present study we observe similar interface damage also for the sputter deposition of ZnO: even if the critical CIGS/AlO_*x*_ interface is made initially virtually defect-free by annealing *before* AZO deposition, detrimental interface defects are present *after* AZO deposition if the passivation layer is too thin to protect the CIGS surface. Similar observations could also apply to different dielectrics. In principle, such additional interface defects created by the ZnO sputter process can be controlled and removed by thermal annealing after AZO deposition. For nanometre-scale front layers, however, this directly leads to the challenges concerning Zn diffusion into the CIGS absorber and potential destruction of the device.

Our measurements thus confirm that sputter damage to the buried CIGS/dielectric interface is a real concern, which becomes increasingly challenging to mitigate if the thickness of the passivation layer is reduced to a few nanometres. These considerations deal with excessive – and thus detrimental – Zn diffusion into the CIGS absorber, which can lead to a reduced long-term stability or sudden failure of the solar cell after heat treatments and thus need to be avoided.

In addition, however, the clear benefits of moderate Cd or Zn diffusion must always be considered in the development of novel front layers or stacks: high dopant concentrations close to 10^17^ cm^−3^ in the absorber layer result in a narrow space charge region (SCR) and strong electric fields close to the front interface. Without the effect of Cd or Zn diffusion reducing the net doping near the absorber surface, the SCR width in a CIGS solar cell at zero bias would only be approximately 100–150 nm. On the one hand, the SCR plays a crucial role in charge collection, and such a narrow *p/n*-junction could lead to significant collection losses. On the other hand, high electric fields inside the junction increase the probability of tunnelling-enhanced interface recombination. Without Cd or Zn in-diffusion, interface recombination could be significantly enhanced by the high electric fields resulting from the high net dopant concentration in the CIGS bulk. This issue could be further magnified by the formation of interface defects during the sputter deposition of the front contacts. Such losses due to field-enhanced interface recombination are in fact highly relevant for solar cells with “Cu-rich” absorbers grown under Cu excess: Despite excellent electronic quality, these “Cu-rich” solar cells suffer from low efficiencies limited by dominant interface recombination due to a high net dopant concentration in the bulk and a defect level caused by the strong KCN etching required to remove secondary phases^58–60^. Without proper control of the CIGS bulk doping achieved by the front layer stack, Cu-poor absorbers with alternative front layers might face the same limitations encountered in “Cu-rich” absorbers today. In fact, it is intriguing to speculate that the higher net dopant concentration within the SCR of “Cu-rich” solar cells, compared to Cu-poor ones, has its origin in a reduced Cd or Zn diffusion due to the lower concentration of Cu vacancies.

Any alternative front layer replacing the CdS buffer layer in a standard solar cell with ZnO front layer must thus, in addition to providing an excellent level of surface passivation and facilitating a correct conduction band offset, have the following additional properties:

1. It must sufficiently block Zn diffusion under environmental conditions encountered during device fabrication and (long-term) operation.
2. If it permits Zn diffusion at elevated temperatures, it must completely shield the CIGS surface from sputter damage during ZnO deposition because post-deposition annealing to recover interface damage might destroy the device.
3. Although excessive impurity diffusion must be avoided, moderate and controlled diffusion from the font layer itself or from ZnO is desired to carefully reduce the net dopant concentration in state-of-the-art CIGS absorber.

The interrelations identified above necessitate a holistic approach to thin-film photovoltaics: the electronic properties inside the absorber layer are largely dictated by chemical diffusion of metal constituents across interfaces in the multi-layered device structure, and are thus directly dependent on processing and post-deposition conditions during solar cell fabrication. The buffer layer or alternative front-passivation layer, for example, does not only control interface recombination, but also promotes or impedes inter-diffusion processes that ultimately determine bulk dopant concentration and density of trap states within the absorber layer. As a result, the relevant electronic properties of the same absorber layer can vary by orders of magnitude even for slightly different front layer materials or processing conditions.

We propose that the main challenge for the development of alternative front contact materials lies in the intricate interrelations between front layer processing and electronic bulk properties in the absorber layer, combined with conflicting requirements for such a suitable front layer: it must protect the CIGS surface from interface damage and long-term degradation, and it must also promote a suitable reduction of the initially high net doping in CIGS without creating recombination-active trap states and without reducing the net doping too much.

## Conclusions

Few-monolayer thin dielectric passivation layers are a promising concept for full-area passivated tunnelling contacts to replace the CdS buffer layer in CIGS solar cells. We demonstrated that even a few monolayers of AlO_*x*_ can be deposited onto rough polycrystalline CIGS surfaces in a highly controlled manner using ALD. After annealing these ultra-thin layers showed excellent interface passivation properties and suitable current transport characteristics for full-area tunnelling contacts. Fabricating complete solar cell devices by depositing a ZnO-based transparent front contact layer onto the AlO_*x*_ tunnelling layer, however, resulted in extremely poor solar cell performance which showed further degradation upon thermal annealing.

Processing conditions of the front contact layers and diffusion of constituent elements across the buffer/absorber interface play a crucial role in defining the electronic junction quality and bulk properties of the CIGS absorber layer. We found that Zn migrates over long distances in CIGS, which significantly reduces – and in extreme cases completely compensates – the net dopant concentration within the CIGS bulk. Literature diffusion studies confirm that such extreme Zn migration is possible, likely facilitated by copper vacancies in CIGS providing a favourable pathway for metal impurities even at low process temperatures.

Although excessive in-diffusion is detrimental and leads to the destruction of the device, some level of Cd- or Zn-diffusion was found to be necessary to reduce the high net doping in state-of-the-art CIGS absorber layers, which otherwise results in an extremely narrow SCR width of only 100–150 nm. Control over surface-near net doping via Zn diffusion was possible with nanoscale AlO_*x*_ passivation layers, but these layers were too thin to shield the CIGS surface from sputter damage during ZnO deposition. Recovering this surface damage by thermal annealing needs further optimization, because the drastically accelerated Zn diffusion needs to be taken into account. No reduction in bulk doping was observed with fully insulating 20 nm thick Al_2_O_3_ interlayers, which apparently blocked all Zn diffusion between ZnO and CIGS.

All these issues pose new constrains on the development of alternative buffer layers or front passivation approaches beyond ensuring low absorptivity and excellent interface quality after full device fabrication: Any alternative to replace CdS also has to carefully control electronic bulk properties by mediating diffusion between CIGS absorber layer and transparent front contact, usually ZnO, under all environmental conditions throughout the lifetime of the device.

## Methods

Cu-poor CIGS thin-film absorbers with a thickness of approximately 2 µm were deposited by co-evaporation on Mo-coated soda-lime glass (SLG) in a three-stage process. The average elemental composition as determined by energy-dispersive x-ray (EDX) corresponds to CGI = [Cu]/([Ga] + [In]) = 0.92–0.98 and GGI = [Ga]/([Ga] + [In]) = 0.27–0.30.The grain size of the polycrystalline films, estimated from SEM top-view images, is 1.1 ± 0.5 µm.

Absorbers for surface passivation are etched in a 5 wt-% aqueous solution of potassium cyanide (KCN) for 30 s to remove surface contaminations, immediately sealed in vacuum bags and shipped to the Institute for Solar Energy Research Hamelin (ISFH) for AlO_*x*_ deposition by plasma-assisted ALD at a substrate set temperature of 130 °C. Reference devices have a standard CdS buffer layer deposited by chemical bath deposition (CBD) for 5–6 min at 67 °C (2 mM CdSO_4_, 50 mM thiourea, 1.5 M NH_4_OH) instead of ALD-AlO_*x*_. The thickness is estimated to be 40–50 nm from typical growth rates.

A ZnO-based transparent front contact (ZnO:Al single layer on AlO_*x*_, i-ZnO/ZnO:Al double layer on CdS) for solar cells is deposited by rf sputtering, and a Ni/Al front contact grid is deposited by electron beam evaporation through a shadow mask.

Solar cells with an active area of 0.1–0.5 cm^2^ are defined by mechanical scribing and non-passivated references achieve efficiencies of 16–18% with CdS/ZnO front stack. Note that no alkali post-deposition treatments or anti-reflection coatings have been applied to any of these devices. Solar cell parameters are determined from current–voltage measurements at room temperature under 1 sun illumination in a class AAA solar simulator. The active device area is determined with an optical microscope and a precision ruler.

Thermal annealing is performed inside a graphite box in a rapid thermal processing (RTP) tool with a background pressure of 800 mbar of nitrogen. A thermocouple probing the temperature of the graphite box is used to control the process.

Capacitance measurements are performed in a closed-cycle cryostat in the dark at a stabilized temperature of 300 K with an LCR meter (frequency range: 20 Hz–2 MHz, ac voltage amplitude: 30 mV rms) assuming a standard parallel equivalent circuit model. For voltage-dependent measurements we monitor the actual dc voltage across the terminals of the MIS device to account for the internal resistance of the LCR meter. Devices are kept at 300 K in the dark at least 12 h before the start of the measurement.

For PL measurements, CIGS/AlO_*x*_ samples are excited by a diode laser at a wavelength of 633 nm at room temperature, and the emitted PL is recorded by an InP/InGaAs near-infrared photomultiplier tube in a wavelength band of ±50 nm around the peak maximum.

## Supplementary information

Supplementary information.
