# Supplementary material for: Ultra-thin passivation layers in Cu(In,Ga)Se2 thin-film solar cells: full-area passivated front contacts and their impact on bulk doping
Source: Sci Rep. 2020 May 5;10:7530. doi: 10.1038/s41598-020-64448-9 (PMC7200765; doi:10.1038/s41598-020-64448-9)
Supplement: Supplementary file 1 — Supplementary information. [file 41598_2020_64448_MOESM1_ESM.pdf]

Supplementary Information

Ultra-thin passivation layers in Cu(In,Ga)Se$_2$ thin-film solar cells: full-area passivated front contacts and their impact on bulk doping

Florian Werner[1], Boris Veith-Wolf[2], Michele Melchiorre[1], Finn Babbe[1,a], Jan Schmidt[2,3], Susanne Siebentritt[1]

1) *University of Luxembourg, Laboratory for Photovoltaics, Department of Physics and Materials Science, 41 rue du Brill, L-4422 Belvaux, Luxembourg.*
2) *Institute for Solar Energy Research Hamelin (ISFH), Am Ohrberg 1, D-31860 Emmerthal, Germany.*
3) *Department of Solar Energy, Institute of Solid-State Physics, Leibniz University Hannover, Appelstr. 2, D-30167 Hannover, Germany.*
a) *now working at Lawrence Berkeley Laboratory, Berkeley, USA.*
*) *corresponding author: fwerner.83@gmx.de*

A: Interface states in admittance spectroscopy

Defects can be identified from steps in the temperature-dependent capacitance spectra (see, e.g., Ref. [S1,S2]). Attribution of individual capacitance features to bulk-like or interface defects is possible based on the voltage dependence of the inflection frequency, for the devices studied here see Ref. [S3] for further details. Capacitance steps at low temperatures not marked in Fig. S1 and S2 are due to mobility or carrier freeze-out, where the full absorber thickness becomes insulating. Capacitance spectra for CIGS/AlO$_x$/metal devices with very thin AlO$_x$ layer are not conclusive, because leakage currents are high without a thick insulating Al_2O_3 layer or a rectifying n^{++} AZO top layer. Figure S1 therefore shows the effect of annealing and AZO deposition on the example of a 20 nm thick Al_2O_3 layer. Figure S2 presents selected spectra for ultrathin AlO$_x$ layers and confirms interface damage due to AZO deposition (left graph) and freeze-out phenomena at room-temperature after annealing in the presence of AZO (right graph). The temperature range in both figures corresponds to a set temperature between 320 K (red, upper curves) and 20 K (blue, lower curves). The measured real sample temperature is approximately 320 – 50 K.

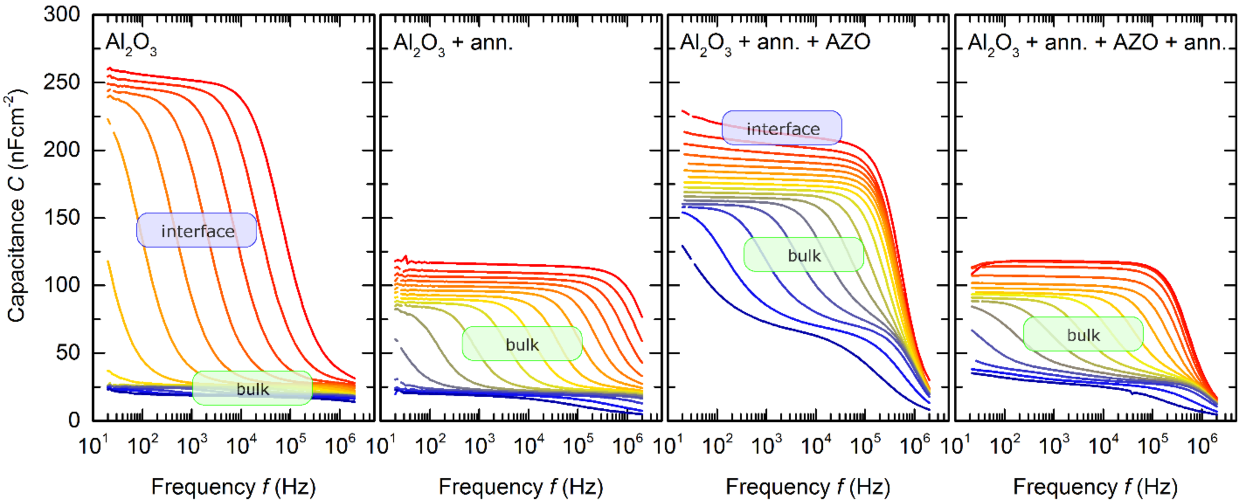

Figure S1: Temperature-dependent capacitance spectra between 320 K (red, upper curves) and 20 K (blue, lower curves) in steps of 20 K set temperature for the same CIGS device passivated with 20 nm of Al_2O_3. Measurements after consecutive processing steps from left to right: as-deposited, annealed for 2.5 min at 350 °C, with AZO layer deposited by rf-sputtering, and after a further annealing step for 1 min at 350 °C.

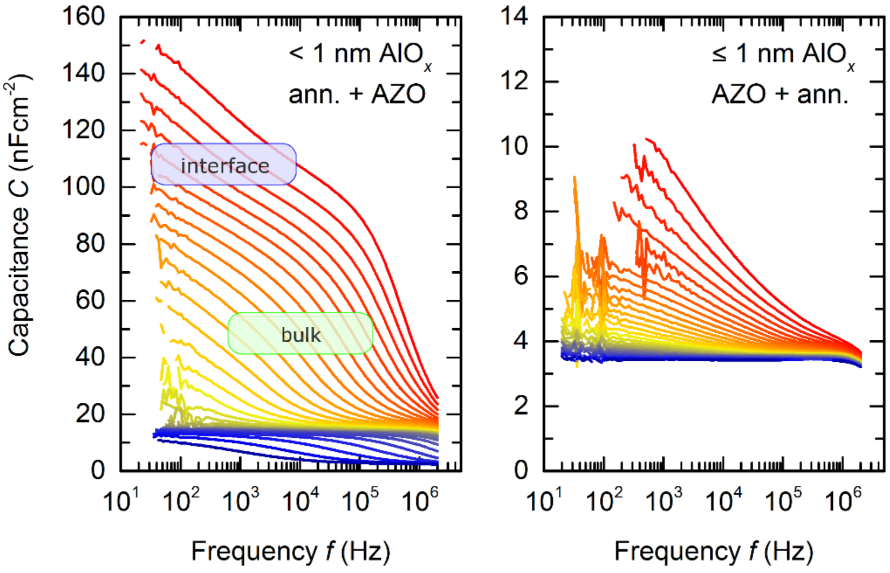

Figure S2: Capacitance spectra for CIGS/AlO_x/AZO devices with thin AlO_x layers between 320–20 K set temperature in steps of 10 K. Left: annealing *before* AZO (CIGS + 7 ALD cycles of AlO_x + annealing for 2.5 min at 350 °C + rf-sputtering of AZO). Right: annealing *after* AZO (CIGS + 8 ALD cycles of AlO_x + rf-sputtering of AZO + annealing for 1 min at 350 °C). Note the different capacitance scale in both graphs.

B: Effect of AlO_x thickness on sputter damage during ZnO deposition

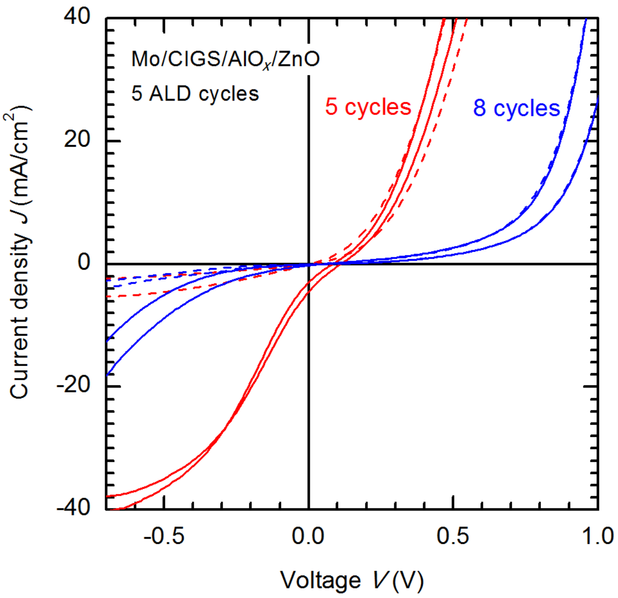

Figure S3: Dark (dashed lines) and illuminated (solid lines) current density – voltage (*JV*) characteristics of as-deposited Mo/CIGS/AlO_x/ZnO solar cells with different thickness of the AlO_x passivation layer: 5 ALD cycles (red lines, approximately 0.5–0.6 nm) and 8 ALD cycles (blue lines, approximately 0.8–1.0 nm), respectively. Two different devices are shown for each thickness.

C: Sodium accumulation at the Al_2O_3/CIGS interface

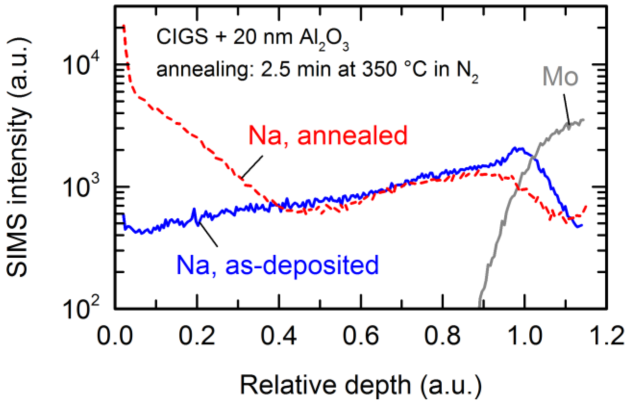

Figure S4: SIMS depth profiles of Na in CIGS passivated with 20 nm of Al_2O_3 before (blue solid line) and after (red dashed line) annealing for 2.5 min at 350 °C. The position of the back contact is estimated from the Mo signal (gray line, only shown for the as-deposited sample).

References:

S1 Nicollian, E. H. & Brews, J. R. *MOS (Metal Oxide Semiconductor) Physics and Technology* (Wiley, 1982).

S2 Blood, P. & Orton, J. W. *The Electrical Characterization of Semiconductors: Majority Carriers and Electron States* (Academic Press, 1992).

S3 Werner, F. *et al.* Oxidation as key mechanism for efficient interface passivation in $Cu(In,Ga)Se_2$ thin-film solar cells. *submitted* (2019).
